# Exploring RNA G‐Quadruplex Stability in the Gas Phase: Insights from Native Mass Spectrometry

**DOI:** 10.1002/cplu.202500679

**Published:** 2025-12-10

**Authors:** Anna Ploner, Sarah Viola Heel, Kathrin Breuker

**Affiliations:** ^1^ Institute of Organic Chemistry and Center for Molecular Biosciences Innsbruck (CMBI) University of Innsbruck Innsbruck Austria

**Keywords:** collisionally activated dissociation, native mass spectrometry, quadruplex, RNA

## Abstract

Native mass spectrometry (MS) of ribonucleic acids (RNA) is a developing technique for the study of RNA structure and interactions that can provide important information on binding stoichiometry and binding motifs. However, the stabilities of key structural elements of RNA, including G‐quadruplex structures, during and after desolvation are not well understood. Efforts to improve our understanding of RNA structures in the gas phase are complicated by the fact that the most common technique for transferring RNA from solution to the gas phase, electrospray ionization (ESI), typically produces ions with a relatively wide distribution of net charges. Here, we have studied two tetramolecular RNA G‐quadruplexes with very different stabilities in solution by native MS. For both K^+^ and ^+^NH_4_ as central cations, we found that the ratio of quadruplex to monomer ions in the ESI spectra was higher for the RNA G‐quadruplex that exhibited higher stability in solution, regardless of ion net charge. These data for native MS of RNA in negative ion mode contrast with data from native MS of proteins in positive ion mode, for which lower and higher net charge is often associated with folded and unfolded structures, respectively. We next probed the stabilities of the gaseous RNA G‐quadruplex ions using collisionally activated dissociation (CAD). For quadruplex ions with K^+^ as central cations, we observed a steady decrease in stability with increasing net charge, consistent with Coulombic repulsion weakening the hydrogen bonding networks and the interactions between the central cations and the nucleobases forming the central channel of the quadruplex. Importantly, for quadruplex ions with the same net charge and for both K^+^ and ^+^NH_4_ as central cations, the RNA G‐quadruplex that exhibited higher stability in solution also demonstrated higher stability in the gas phase. Furthermore, we demonstrate that for quadruplexes with lower net charge and K+ as central cations, covalent bond cleavage occurs at lower energies available for dissociation than strand separation.

## Graphical abstract

This study explores RNA G‐quadruplex structures using native mass spectrometry to better understand their stability during desolvation and in the gas phase. Although increasing net charge reduces quadruplex stability due to Coulombic repulsion, even highly charged quadruplex ions do not spontaneously dissociate. Notably, for quadruplexes with lower net charge, covalent bond cleavage occurs at lower energies than strand separation.

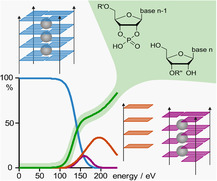

## 1 Introduction

Ever since the introduction of electrospray ionization (ESI) for mass spectrometry (MS) of larger biomolecules [1, 2, 3, 4, 5, 6], and the subsequent discovery that ESI can preserve intermolecular noncovalent interactions during and after transfer into the gas phase [7, 8, 9, 10, 11, 12], researchers have been speculating about the stability of solution structures in the absence of solvent water [13]. Then, experimental studies of proteins [14, 15, 16, 17, 18, 19, 20] provided strong support for the idea that removing the aqueous environment can cause substantial structural changes [21]. These studies also revealed that the regions of a solution structure that are most stable after transfer into the gas phase are those stabilized by salt bridges and ionic hydrogen bonds. In line with these observations, it was later found that in the gas phase, the electrostatic interactions between ribonucleic acids (RNA) and positively charged peptides or aminoglycosides can be more stable than the covalent backbone bonds of the RNA [22], allowing for binding site mapping in top–down MS experiments [23, 24, 25, 26, 27]. However, the stability of RNA structures in the gas phase remains poorly understood, especially when these negatively charged structures are not stabilized by a positively charged binding partner, or when the stabilizing positive charge is a small cation such as K^+^ in G‐quadruplex structures [28, 29, 30, 31, 32, 33]. Here, we report on the stability of tetramolecular G‐quadruplex structures in the gas phase, focusing on how the identity of the central cations and the net charge of the quadruplex ions affect both the energies required for dissociation and the dissociation pathways, i.e., strand separation versus covalent bond cleavage.

Gaseous ions of RNA and DNA (deoxyribonucleic acid) G‐quadruplexes have been investigated in numerous studies [34, 35]. The G‐quadruplexes were monomolecular [36, 37], bimolecular [38], or tetramolecular [39, 40, 41, 42, 43] assemblies of RNA or DNA with different central cations, primarily K^+^ and ^+^NH_4_, and in most cases produced by ESI in negative ion mode. The mechanism of ESI, especially the factors that determine the net charge of ions, is not well understood [44, 45, 46, 47, 48]. An intriguing and frequently discussed phenomenon of ESI is that it produces ions in multiple charge states, meaning that ions of a given RNA or RNA assembly are typically observed with a distribution of net charges. For example, the gaseous (M ‐ nH)^n−^ ions from ESI of an RNA typically exhibit a relatively broad distribution of net charge values n, which can be manipulated by the use of ESI additives [46]. Likewise, ions of 24 nt (nucleotide) DNA G‐quadruplexes, electrosprayed from common biochemical buffers using special “nanopore ion emitters”, were observed in a wide m/z range (550–1100), with net charge values n ranging from 7 to 14 [36]. ESI of G‐quadruplexes in solutions containing trimethylammonium acetate [42] or ammonium acetate [49, 50, 51], which can provide ionic strength but lack buffering capacity at physiological pH [52], produces ions with fewer net charges per nucleotide. Although the mechanism by which ions are produced in ESI is still unclear, the fact that the net charge values n are generally smaller than the number of phosphodiester moieties of a given RNA, RNA complex, or RNA assembly is a clear indication of proton transfer from solvent and/or additive molecules or ions to the RNA anions during ESI. Proton transfer also plays a role in the dissociation of gaseous noncovalent complexes of RNA and small molecule ligands [53], including peptides [22] and aminoglycosides [25]. Specifically, proton transfer resulting from vibrational activation can convert salt bridges between protonated ligands and deprotonated RNA into far weaker hydrogen bonds that more easily break, causing RNA and ligand to dissociate [53]. Because G‐quadruplex structures in living systems are stabilized by interactions with positively charged ions that cannot transfer a proton, i.e., K^+^ or Na^+^, we wondered about the strength of these noncovalent interactions in the gas phase, and whether they could be stronger than the covalent bonds of the RNA. To address this question, we have studied two tetramolecular RNA quadruplexes of very different stability, both with K^+^ or ^+^NH_4_ as central cations. K^+^ and ^+^NH_4_ have similar radii “of the cavity formed by the ion in a particular solvent” [54], but unlike K^+^, ^+^NH_4_ is capable of proton transfer.

## 2 Experimental

RNA was electrosprayed at a flow rate of 5.0 µl/min in negative ion mode from solutions containing 2 µM RNA in H_2_O with different ESI additives, as described in the main text. H_2_O was purified to 18 MΩ·cm at room temperature using a Milli‐Q system (Millipore, Austria). RNAs **1** and **2** (Table 1), ammonium bicarbonate (≥99.0%), ammonium acetate (≥99.0 %), potassium chloride (≥99.0%), triethylammonium acetate (TEAA, 1.0 M in H_2_O at pH 7), and 2,2‐difluoroethylamine (DFEA) [55] were from Sigma Aldrich (Austria), and 1,1,1,3,3,3‐hexafluoroisopropanol [56] (HFIP, >99.5%) was from Fluorochem (England). The purity and integrity of the RNAs were confirmed by ESI MS. RNA concentration was determined by UV absorption at 260 nm using an Implen Nano Photometer (Implen, Germany). The pH of the solutions for ESI was measured using nonbleeding pH indicator strips (Merck, Germany) and the solutions were used without further pH adjustment. For solutions containing K^+^, we found that the highest concentration of KCl for obtaining ESI spectra of quadruplex ions was 120 μM; above this concentration, extensive (KCl)_*n*_·Cl^−^ cluster ion formation along with RNA signal suppression was observed. RNA solutions for ESI were prepared from stock solutions in H_2_O (RNA **1**: 154 μM, RNA **2**: 95 μM, stored at −20°C) and electrosprayed after ≈10 min; increasing this time to up to 60 min had no effect on the relative heights of the signals of different RNA species (monomeric, dimeric, and tetrameric) observed in the spectra. We also attempted to study quadruplexes with Na^+^ as central cations by electrospraying RNA **1** from solutions containing 120 μM NaCl and no added KCl. However, ESI of these solutions did not produce any quadruplexes with exclusively Na^+^ as central cations but instead produced quadruplexes with both Na^+^ and K^+^, suggesting a higher affinity for K^+^ over Na^+^, both of which are ubiquitous and cannot be completely eliminated from the RNA samples.

**TABLE 1:** RNA studied.

RNA	Sequence^a^	nt	Elemental composition	M_measured_ b	M_calculated_ b	T_m_ (quadruplex)/°C^c^
**1**	UAGGGU	6	C_58_H_71_N_24_O_41_P_5_	1914.289	1914.290	79.0
**2**	UUAGGG	6	C_58_H_71_N_24_O_41_P_5_	1914.283	1914.290	49.1

a From 5′‐OH to 3′‐OH terminus;

b M refers to monoisotopic mass in Da,

c Melting temperatures T_m_ from reference [41].

MS experiments were performed on a Thermo Scientific Orbitrap Fusion Lumos Tribrid mass spectrometer equipped with an ESI source, a linear quadrupole for isolation of the ions of interest, and an ion‐routing multipole floated with N_2_ gas for vibrational activation by collisionally activated dissociation (CAD). A spray voltage of 3400 V (solutions containing K^+^) or 3500 V (solutions containing ^+^NH_4_) was used for ESI. The spray voltage and other instrument settings were optimized for maximum quadruplex ion signals, as detailed in the following: 275°C (“ion transfer tube”), 60°C (“vap temperature”), 32 (“sheath gas” in arbitrary units), 0.2 and 0.5 (“aux gas” in arbitrary units for solutions containing K^+^ and ^+^NH_4_, respectively), 0 (“sweep gas” in arbitrary units), 150% (“RF lens”), 1000 (“AGC target”), 1000 ms (“injection time”). The laboratory‐frame collision energy for CAD (“activation type”: HCD, “CE mode”: fixed, “CE type” absolute) was adjusted by varying the “HCD CE energy” values from 0 V to 60 V. This parameter represents a potential (measured in volts) rather than an energy; laboratory‐frame collision energies were calculated by multiplying the values in volts with the absolute net charge of the ions. As discussed in ref. [57] and [53], calibration of a laboratory‐frame collision energy scale to an internal energy scale in CAD experiments under multiple‐collision conditions is not straightforward. However, the effects of degrees of freedom (DOF) are marginal because the atomic composition of all quadruplex ions of RNAs **1** and **2** studied is the same except for the number of deprotonation sites (introducing a <2% variation in the number of atoms for quadruplexes with K^+^) and the type of central cations (introducing a ∼4% difference in the number of atoms between quadruplexes with K^+^ and ^+^NH_4_). Furthermore, because the quadruplex ions were studied under the same experimental conditions on the same instrument, we can draw meaningful conclusions from our data, despite the energy scale not being absolute. Twenty‐five scans (for accurate mass measurements) or 50 scans (for CAD) were added for each spectrum. Data reduction utilized the programs Freestyle (Thermo Fischer, Austria), mMass written by Martin Strohalm (https://github.com/xxao/mMass) [58], and FAST MS, which was programmed in our group by Michael Palasser (https://github.com/michael‐palasser/FAST‐MS/releases) [59], as well as manual inspection of the spectra.

UV melting profiles were recorded at 250, 260, and 295 nm on a Varian Cary‐100 spectrophotometer equipped with a multiple cell holder and a Peltier temperature control device. RNA samples for UV melting experiments were prepared as 2 μM or 95 μM solutions with potassium chloride (120 μM) and 200 mM HFIP, and transferred into 800 or 330 μl UV‐permeable high‐precision cells made of quartz SUPRASIL with a light path of 10 mm or 1 mm, respectively. UV melting profiles were recorded with a heating and a subsequent cooling ramp at a rate of 0.7°C/min for 2 μM and 0.2°C/min for 95 μM RNA concentrations.

## 3 Results and Discussion

The 6 nt sequences of RNA **1** and **2** (Table 1) are derived from human telomeric repeat‐containing RNA, which are transcription products of telomeres. Experiments by Komiyama and coworkers using nuclear magnetic resonance (NMR) spectroscopy, circular dichroism (CD) spectroscopy, and nondenaturing polyacrylamide gel electrophoresis (PAGE) showed that both RNA **1** (5′‐UAGGGU‐3′) and **2** (5′‐UUAGGG‐3′) form tetrameric G‐quadruplex structures in solutions with K^+^ or Na^+^ at room temperature [41]. Moreover, CD melting experiments revealed a far higher stability of the quadruplex structure of RNA **1** (melting temperature T_m_ = 79°C; Δ*G* = −26.2 kJ/mol at 37°C) compared to that of RNA **2** (T_m_ = 49°C; DG = −4.2 kJ/mol at 37°C), which was attributed to stabilization by the 3′‐terminal U‐tetrad of RNA **1** [41]. To address the question of whether the solution structures of the tetrameric quadruplexes of RNA **1** and **2** can be preserved during and after removal of the aqueous environment and to probe their stability in the gas phase, we studied RNA **1** and **2** by native ESI MS and CAD.

**FIGURE 1:**
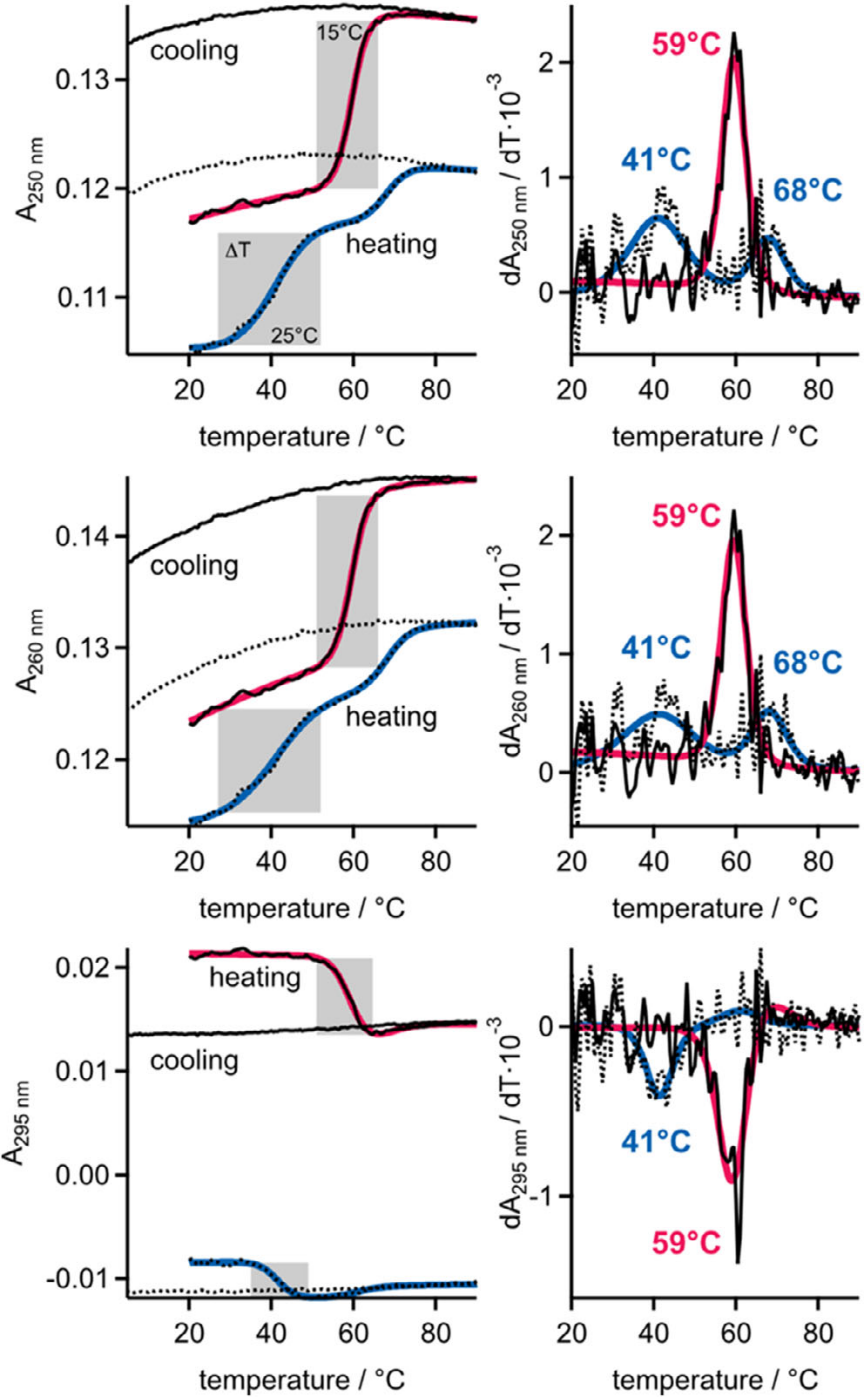
UV absorption, A, and dA/dT at 250, 260, and 295 nm of aqueous solutions of RNA **1** (solid black lines) and RNA **2** (dashed black lines) with potassium chloride (120 µM) and 200 mM HFIP (pH 5.5) as used for ESI experiments, RNA concentration was 2 µM. The heating curves were empirically found to fit functions containing a linear term in T and one or two sigmoidal terms in T; these fits and their derivatives are overlaid on the heating curves and their derivatives in red for RNA **1** and blue for RNA **2**.

ESI can transfer RNA from solution into the gas phase, but the solutions used in the above CD melting experiments are unsuitable for conventional ESI due to the high concentrations of nonvolatile salts (150 mM NaCl, 10 mM sodium phosphate). To confirm that the G‐quadruplexes of RNA **1** are also more stable than those of RNA **2** in the specific solutions used for ESI here, we performed UV melting experiments with solutions containing 2 µM RNA **1** or RNA **2**, 200 mM HFIP, and 120 µM KCl. Figure 1 shows the UV absorption of these solutions as a function of temperature for both heating and subsequent cooling at a rate of 0.7°C/min. The increase in absorption at both 260 nm and 250 nm and the decrease at 295 nm with increasing temperature (“heating curve”) reflect the dissociation of the G‐quadruplexes. However, at these relatively low RNA concentrations, the association reaction (monitored as the “cooling curve”) was too slow for the heating curve to be reproduced by the cooling curve. In other words, dissociation and association of G‐quadruplexes in these UV melting experiments did not occur at equilibrium, preventing the derivation of thermodynamic quantities from this data, a phenomenon described previously [60]. In an attempt to find conditions under which equilibrium is established, we increased the RNA concentrations to 95 µM and decreased the heating and cooling rates to 0.2°C/min, but this did not significantly accelerate the association reaction (Figure S1). Nevertheless, at both RNA concentrations and all wavelengths studied, the “heating curves” and their derivatives indicate a structural transition at ∼60°C for RNA **1** and one at ∼40°C for RNA **2** (Figure 1 and S1). In addition, the transition for RNA **1** at ∼60°C occurs over a narrower temperature range (Δ*T* ∼ 15°C) than the transition for RNA **2** at ∼40°C (ΔT ∼ 25°C). These findings align with the data from Komiyama and coworkers [41] and demonstrate that the G‐quadruplexes of RNA **1** are more stable than those of RNA **2** in the solutions used for ESI at room temperature [61]. Interestingly, for RNA **2** and at 250 nm and 260 nm but not 295 nm, a second transition was observed at ∼70°C that may suggest a sequential dissociation process of G‐quadruplexes involving dimeric RNA species, consistent with the proposed sequential association mechanism [62].

**FIGURE 2:**
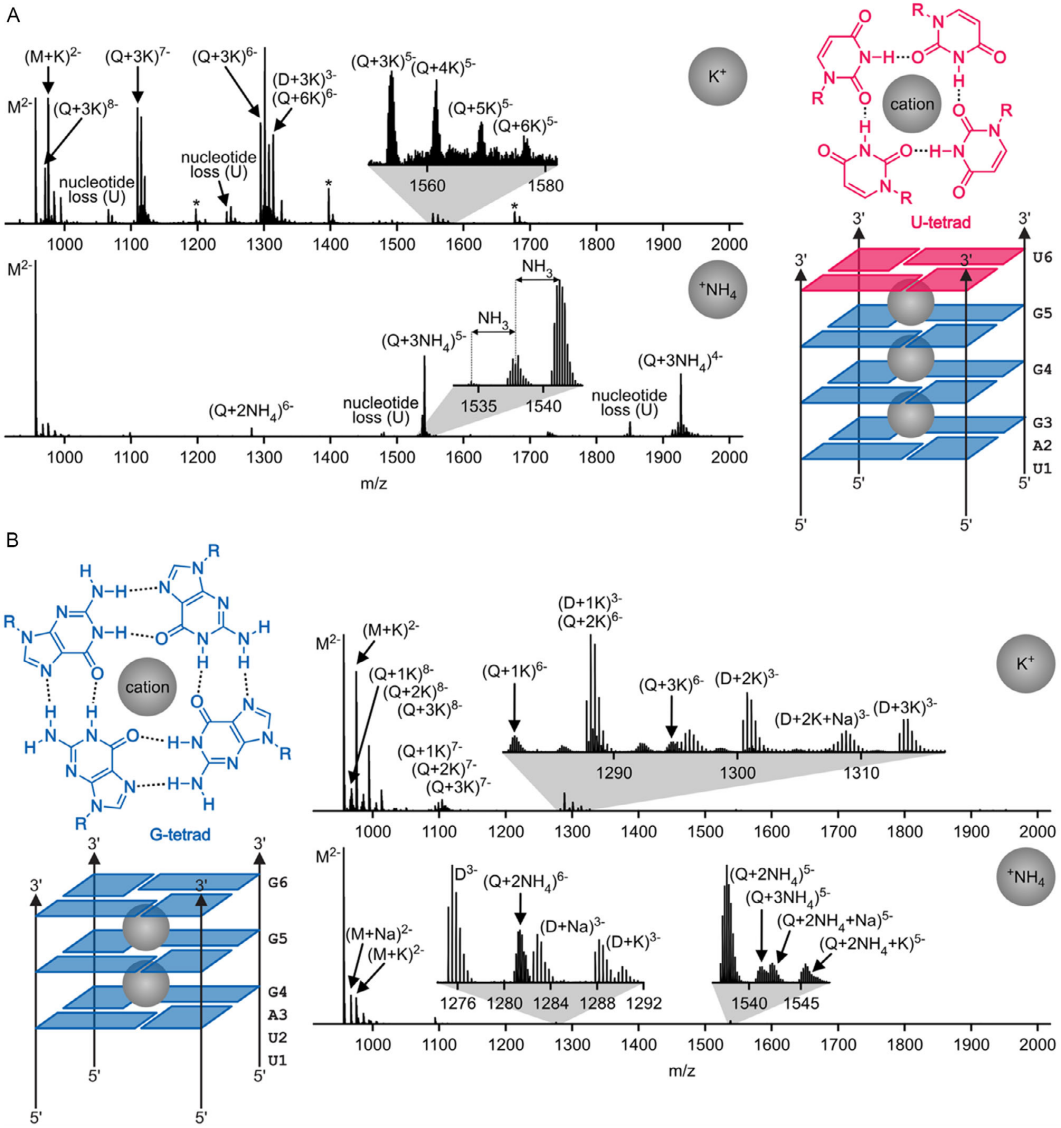
MS spectra of (A) RNA **1** and (B) RNA **2** electrosprayed from aqueous solutions with potassium chloride (120 µM) and 200 mM HFIP (pH 5.5, spectra labeled with K^+^) and 50 mM ammonium acetate and 5 mM ammonium bicarbonate (pH 6.5, spectra labeled with ^+^NH_4_), RNA concentration was 2 µM.

The spectra of RNA **1** electrosprayed from aqueous solutions containing potassium chloride and HFIP (to increase signal intensity without affecting quadruplex integrity in solution) [56] (Figure 2A) showed signals corresponding to tetrameric ions (Table 2) with at least three bound K^+^ ions, in support of the hypothesis that the 3′‐terminal U‐tetrad allows for binding of a third cation within the central channel of the G‐quadruplex of RNA **1** [41]. A similar pattern with three bound ^+^NH_4_ ions emerged when RNA **1** was electrosprayed from aqueous solutions containing ammonium acetate and ammonium bicarbonate at similar pH. However, loss of NH_3_ from tetrameric ions with three bound ^+^NH_4_ ions was also observed, especially for ions with higher net charge (Figure 2A). This is consistent with some unintended vibrational ion activation in the source region of the mass spectrometer, which generally increases with increasing ion net charge, and which also explains the observed loss of U nucleotide. Nevertheless, the spectra of RNA **1** in Figure 2A showed predominantly tetrameric ions with three K^+^ or ^+^NH_4_ ions, which we interpret as quadruplex ions. Tetrameric ions of RNA **1** with more than three K^+^ ions are interpreted as quadruplex ions, in which additional K^+^ ions are attached to deprotonated phosphodiester moieties, and those with two ^+^NH_4_ ions are interpreted as quadruplex ions that have lost NH_3_.

**TABLE 2:** Calculated monoisotopic m/z values for (4·RNA + x·K^+^ − h·H^+^)^n−^ and (4·RNA + x·NH_4_^+^ − h·H^+^)^n−^ ions of RNAs 1 and 2 (which have the same elemental composition) for *x* = 1–3 and *n* = 4–8. To improve readability, the term “h·H^+^” is omitted and “4·RNA” is replaced by “Q” for ion designations in the main text, e.g., (Q + 3K)^n−^ stands for (4·RNA + 3K^+^ − h·H^+^)^n−^.

**(4** **×** **RNA + 3K^+^ − h** **×** **H^+^)^n−^** **= (Q + 3K)^n−^**			**(4** **×** **RNA + 2K^+^ − h** **×** **H^+^)^n−^** **= (Q + 2K)^n−^**			**(4** **×** **RNA + 1K^+^ − h** **×** **H^+^)^n−^** **= (Q + 1K)^n−^**		
n	h	m/z_monoisotopic_	n	h	m/z_monoisotopic_	n	h	m/z_monoisotopic_
4	7	1941.749	4	6	1932.260	4	5	1922.771
5	8	1553.198	5	7	1545.607	5	6	1538.016
6	9	1294.164	6	8	1287.838	6	7	1281.512
7	10	1109.139	7	9	1103.717	7	8	1098.295
8	11	970.371	8	10	965.627	8	9	960.882
**(4** **×** **RNA + 3NH_4_^+^ − h** **×** **H^+^)^n−^** **= (Q + 3NH_4_)^n−^**			**(4** **×** **RNA + 2NH_4_^+^ − h** **×** **H^+^)^n−^** **= (Q + 2NH_4_)^n−^**			**(4** **×** **RNA + 1NH_4_^+^ − h** **×** **H^+^)^n−^** **= (Q + 1NH_4_)^n−^**		
n	h	m/z_monoisotopic_	n	h	m/z_monoisotopic_	n	h	m/z_monoisotopic_
4	7	1926.052	4	6	1921.796	4	5	1917.539
5	8	1540.640	5	7	1537.235	5	6	1533.830
6	9	1283.699	6	8	1280.861	6	7	1278.024
7	10	1100.170	7	9	1097.737	7	8	1095.305
8	11	962.523	8	10	960.394	8	9	958.266

**FIGURE 3:**
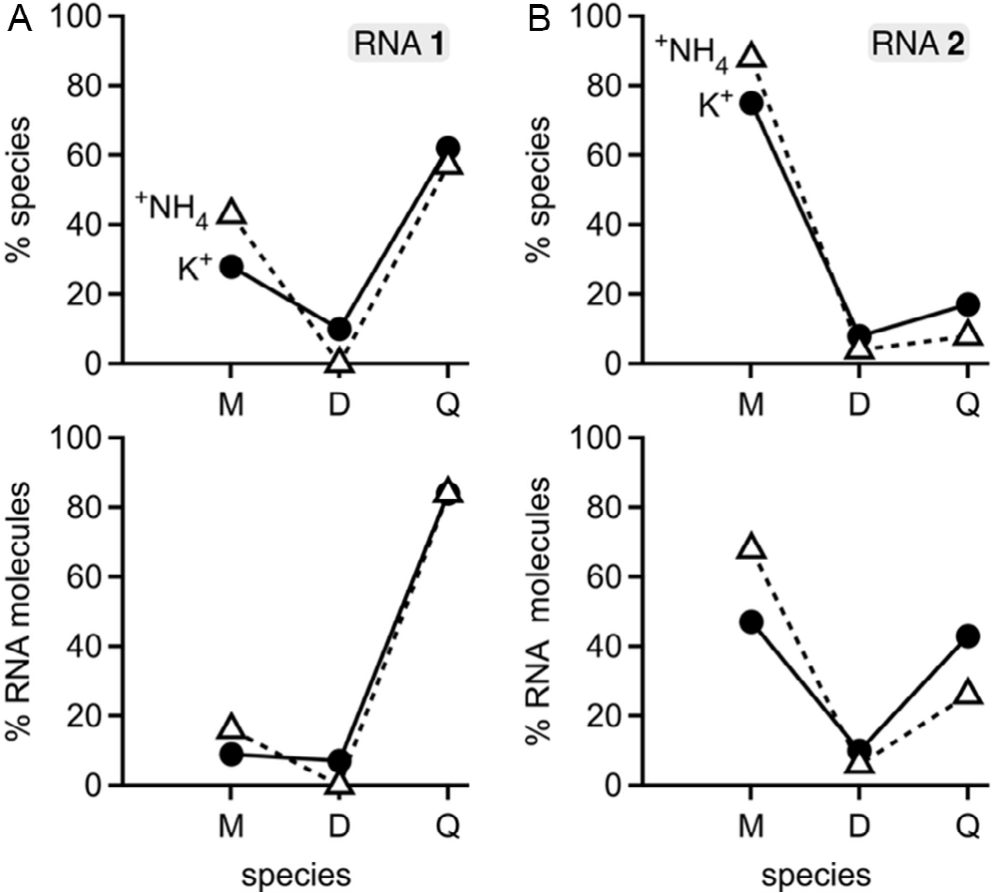
Percentage of monomeric (M), dimeric (D), and tetrameric (Q) RNA species (top) and percentage of RNA molecules in the respective species from the ESI spectra in Figure 2 for (A) RNA **1** and (B) RNA **2**; trimeric species were not observed. Data from ESI of solutions with K^+^ and ^+^NH_4_ are shown as circles and triangles, respectively.

Apart from quadruplex ions (62% for K^+^, 57% for ^+^NH_4_), we observed monomeric (28% for K^+^, 43% for ^+^NH_4_) and dimeric (10% for K^+^, 0% for ^+^NH_4_) species of RNA **1** (Figure 3A, Table S1). As suggested above, the dimeric species may play a role in the association and dissociation of these tetramolecular quadruplexes as an intermediate. Considering that the quadruplexes studied here consist of four RNA molecules, and dimer ions consist of two, the percentage of RNA molecules in the quadruplex ions from ESI was 84% for both K^+^ and ^+^NH_4_ (Figure 3A). Using different ESI solution additives to increase ionic strength (ammonium acetate and ammonium bicarbonate, DFEA, TEAA) produced quadruplex ions with different distributions of net charge (Figure 2 and S2), but they had only a small effect on the percentage of RNA molecules in the gaseous quadruplex ions of RNA **1**. While the ESI process and/or unintended ion activation in the source region of the mass spectrometer can result in some dissociation, the majority of the quadruplexes of RNA **1** withstand transfer from solution into the gas phase, regardless of whether the central cations are K^+^ or ^+^NH_4_, or which net charge they carry.

The spectra of RNA **2** electrosprayed from aqueous solutions containing potassium chloride or ammonium acetate and ammonium bicarbonate (Figure 2B) showed predominantly signals corresponding to monomeric species, in agreement with the far lower stability of the quadruplex structure of RNA **2** compared to that of RNA **1**. Quadruplex ions of RNA **2** were observed with 1–3 K^+^ or 2–3 ^+^NH_4_, but those with two K^+^ or two ^+^NH_4_ were the most abundant, consistent with two central cations sandwiched between the three G‐tetrads. The percentages of RNA molecules in the quadruplexes of RNA **2** with K^+^ (43%) and ^+^NH_4_ (26%) indicate a somewhat lower stability during transfer into the gas phase for the quadruplexes with ^+^NH_4_ as central cations (Figure 3A). Overall, the native ESI MS spectra of RNA **1** and **2** qualitatively reflect the monomer‐quadruplex equilibrium present in solution.

**FIGURE 4:**
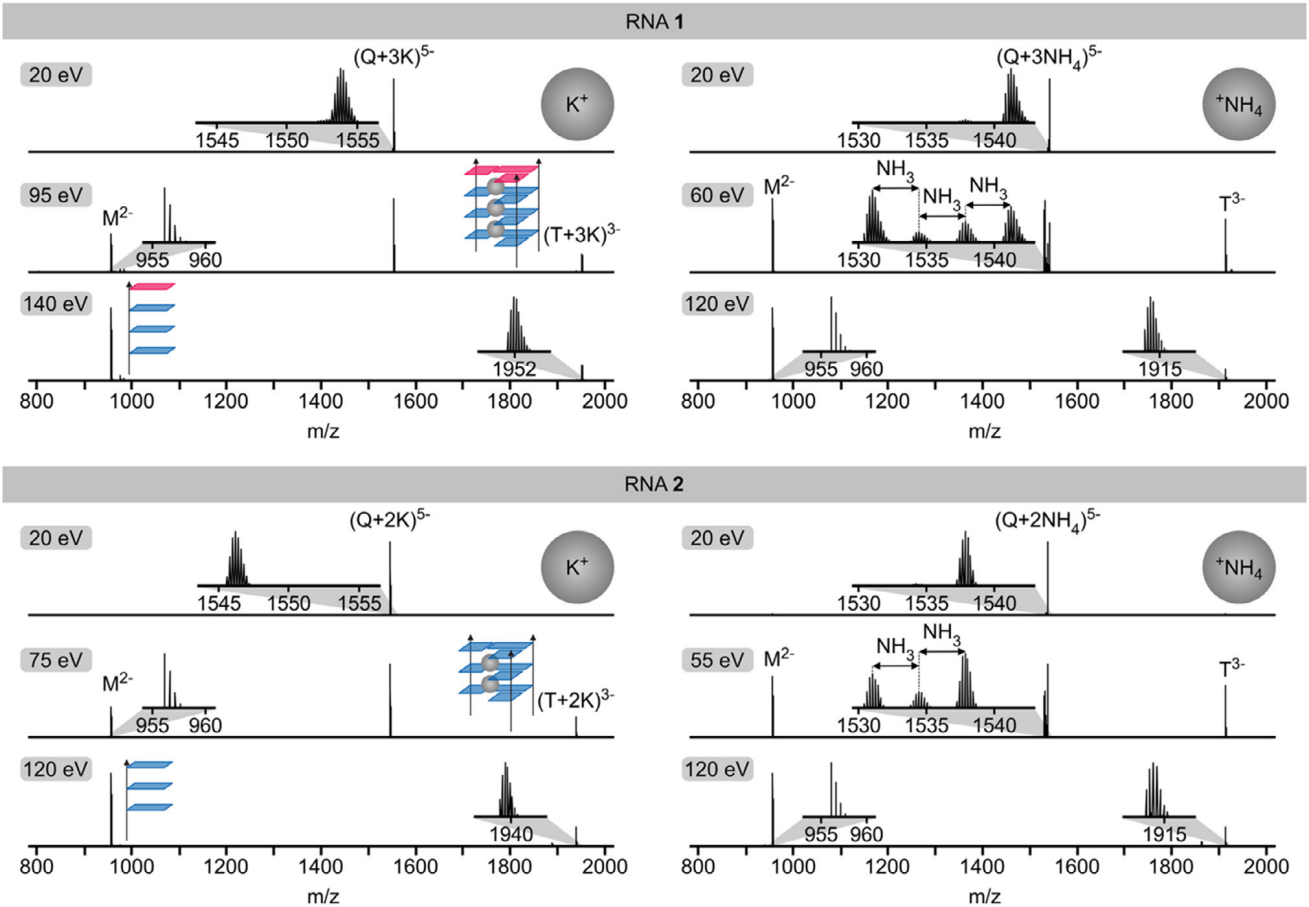
CAD spectra of (Q + 3K)^5−^ and (Q + 3NH_4_)^5−^ ions of RNA **1** and (Q + 2K)^5−^ and (Q + 2NH_4_)^5−^ of RNA **2** using the laboratory‐frame energies indicated in each panel illustrate dissociation into monomeric (M) and trimeric (T) species, and loss of NH_3_ for the quadruplexes with ^+^NH_4_ as central cations.

**FIGURE 5:**
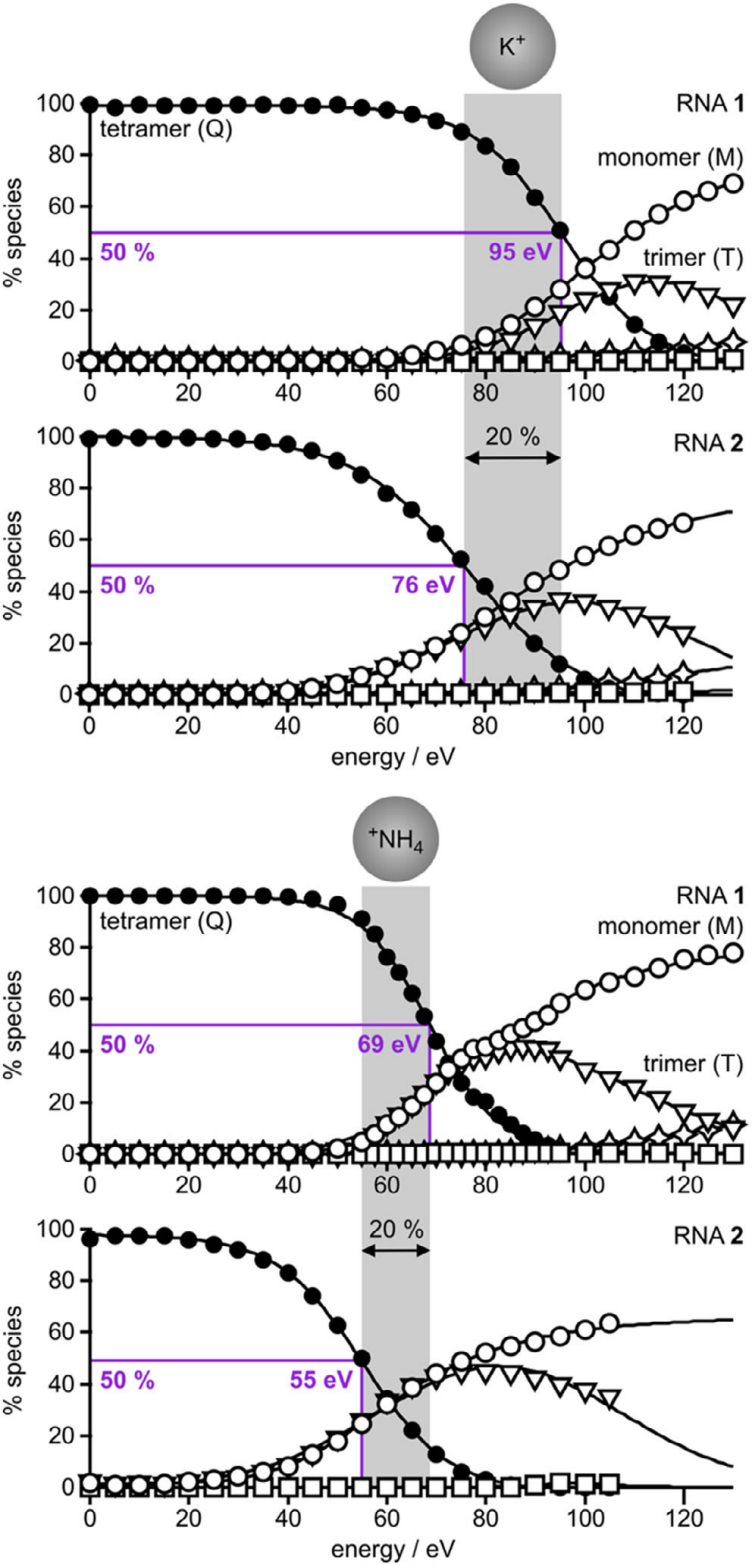
Percentage of monomeric (M, open circles), dimeric (D, squares), trimeric (T, triangles), and tetrameric (Q, solid circles) RNA species from CAD of (Q + 3K)^5−^ and (Q + 3NH_4_)^5−^ ions of RNA **1** and (Q + 2K)^5−^ and (Q + 2NH_4_)^5−^ of RNA **2** versus laboratory‐frame energy; violet lines indicate energies required for 50% quadruplex dissociation (which are 20% higher for RNA **1** than RNA **2** for both K^+^ and ^+^NH_4_ as central cations). Quadruplex ions showing loss of up to three (RNA **1**) or two (RNA **2**) NH_3_ molecules are included in the percentage of tetrameric RNA species. RNA fragment ions from covalent bond cleavage (stars, up to ∼11%) and dimeric species (<2%) were the least abundant of all dissociation products and were only observed at the highest energies used.

As indicated in the title, the key focus of our study was to investigate the stability of tetramolecular RNA G‐quadruplex structures in the gas phase. Figure 4 shows spectra from CAD of (Q + 3K)^5−^ and (Q + 3NH_4_)^5−^ ions of RNA **1** and (Q + 2K)^5−^ and (Q + 2NH_4_)^5−^ ions of RNA **2** at different laboratory‐frame energies available for dissociation. The dominant dissociation pathway for these quadruplex ions was asymmetric dissociation into monomeric M^2−^ and trimeric (T + 3K)^3−^, (T + 2K)^3−^, or T^3−^ ions. Asymmetric dissociation into monomeric and trimeric species, Q → T + M, involves breaking of the same number of hydrogen bonds as symmetric dissociation into two dimeric species: four hydrogen bonds in each G‐tetrad and two in the U‐tetrad (Figure 2). However, asymmetric dissociation generally preserves a larger number of interactions between the central cations and the nucleobases than symmetric dissociation. For example, the two K^+^ ions in the (Q + 2K)^5−^ ions of RNA **2** interact with 12 guanosine bases (Figure 2). Asymmetric dissociation into (T + 2K)^3−^ and M^2−^ ions preserves interactions of the two K^+^ ions with nine bases, while symmetric dissociation into (D + 2K)^3−^ and D^2−^ ions would preserve interactions with only six bases. This should make asymmetric dissociation the lower‐energy pathway for quadruplex dissociation, in agreement with the data in Figure 4 and the fact that dimeric species only appeared at relatively high energies, with yields of no more than 2% (Figure 5).

The breakdown curves in Figure 5 show that in the gas phase, the quadruplexes of RNA **1** and RNA **2** with a net negative charge of five and K^+^ as central cations are more stable (by ∼28%) than those with ^+^NH_4_. This can be attributed to the sequential loss of three (RNA **1**) or two (RNA **2**) NH_3_ molecules at energies lower than those required for quadruplex dissociation (Figure S3). Loss of NH_3_, which was also observed with quadruplexes of DNA [63, 64], involves proton transfer from ^+^NH_4_ to the RNA, most likely to the carbonyl oxygens of guanosine bases in the central channel of the quadruplex, with which ^+^NH_4_ forms hydrogen bonds [65]. Despite the weakening of the quadruplex structure caused by the loss of NH_3_ and thus the central cations, asymmetric dissociation into monomeric and trimeric species was still observed (Figures 4 and 5). This suggests that the trimeric species are stabilized by an extended hydrogen bonding network [22] that includes both positively (protonated guanosine bases) and negatively (deprotonated phosphodiester moieties) charged sites. In agreement with this hypothesis, trimer dissociation into monomers was observed only at elevated energy. Finally, the quadruplexes of RNA **1** were found to be more stable by ∼20% than those of RNA **2**, regardless of whether the central cations were K^+^ or ^+^NH_4_, which reflects the contribution of the U‐tetrad and the additional central cation to overall quadruplex stability.

**FIGURE 6:**
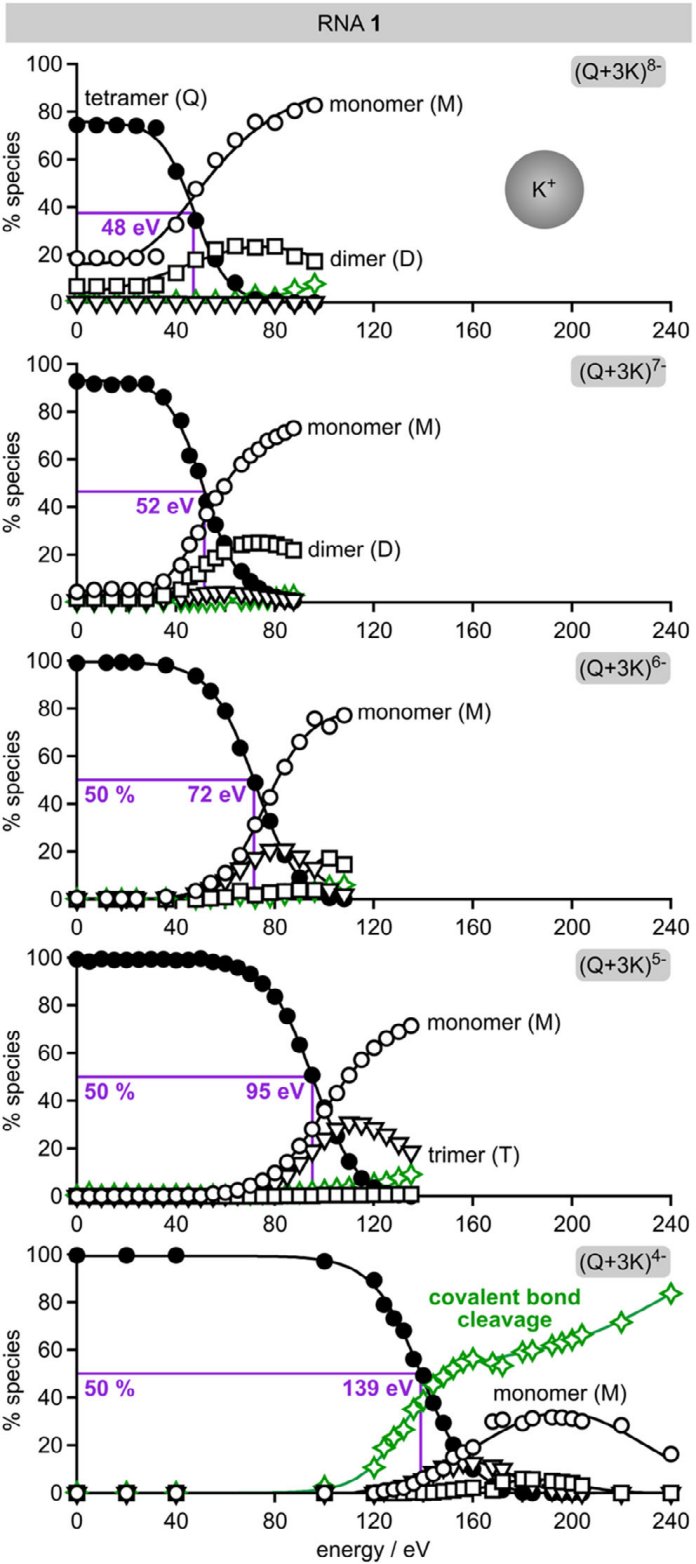
Percentage of monomeric (M, open circles), dimeric (D, squares), trimeric (T, triangles), and tetrameric (Q, solid circles) RNA species, and products from covalent bond cleavage (stars), from CAD of (Q + 3K)^n−^ ions of RNA **1** for *n* = 4–8 versus laboratory‐frame energy; violet lines indicate energies required for 50% quadruplex dissociation. For *n* = 7–8 and 0–30 eV, the percentage of quadruplex ions was less than 100% due to unintended vibrational excitation in the quadrupole used for ion isolation [53], accordingly, the energies required to dissociate half of the quadruplexes were less than 50% of all species.

Given that both positive and negative charges contribute to the stability of the gaseous structures of (Q + 3K)^5−^ and (Q + 3NH_4_)^5−^ ions of RNA **1** and the (Q + 2K)^5−^ and (Q + 2NH_4_)^5−^ ions of RNA **2**, we wondered how changing the negative net charge *n* across a wide range would affect quadruplex stability. As shown in Figure 6 for (Q + 3K)^n−^ ions of RNA **1**, increasing the number of negative charges from 7 (for *n* = 4) to 8 (for *n* = 5) to 9 (for *n* = 6) to 10 (for *n* = 7) to 11 (for *n* = 8) leads to a steady decrease in the energy required for 50% quadruplex dissociation (Figure S4) and thus quadruplex stability in the gas phase. This observation is consistent with the weakening of hydrogen bonding networks and the interactions between K^+^ and the nucleobases in the central channel of the quadruplex by Coulombic repulsion, despite the stabilizing interactions between opposite charges.

Furthermore, the breakdown curves of (Q + 3K)^4−^, (Q + 3K)^5−^, (Q + 3NH_4_)^5−^, (Q + 3K)^6−^, and (Q + 3NH_4_)^6−^ show a parallel increase in the percentages of monomeric and trimeric species at lower energies, which is clear evidence that dissociation into trimeric and monomeric RNA species, Q → T + M, is the lowest‐energy channel for noncovalent bond dissociation (Figures 6 and S4), followed by dissociation of the trimeric into monomeric species at elevated energies. For the highly charged (Q + 3K)^7−^ and (Q + 3K)^8−^ ions, mostly monomeric and dimeric species and only very few trimeric species were detected, indicating a significantly decreased stability of the trimeric species and its rapid dissociation into monomeric and dimeric species, T → D + M. Apparently, increasing the number of deprotonated sites on the RNA backbone also weakens the stabilizing interactions between the central cations and the nucleobases such that dimeric species become more stable than trimeric species during dissociation of the more highly charged (Q + 3K)^7−^ and (Q + 3K)^8−^ ions.

**FIGURE 7:**
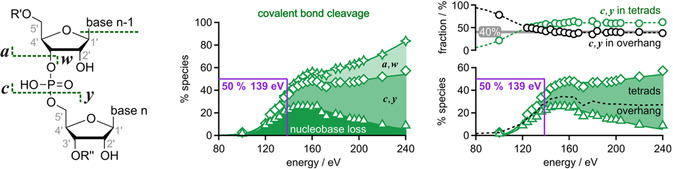
Types of products from covalent bond cleavage in CAD of (Q + 3K)^4−^ ions of RNA **1** were ***c*** and complementary ***y*** fragments, ***a*** and complementary ***w*** fragments, and those originating from nucleobase loss (left). Percentage stacked area plots illustrate how the contribution of each type to the total yield of products from covalent bond cleavage varies with laboratory‐frame energy (center). Separating ***c*** and ***y*** fragments as originating from RNA phosphodiester backbone cleavage between tetrads (***c***_3_ ‐ ***c***_5_ and ***y***_2_; ***y***_1_ is generally uncharged for RNA with an 3′ OH terminus and cannot be detected by MS) and RNA phosphodiester backbone cleavage in the overhang (***c***_1_ ‐ ***c***_2_ and ***y***_4_ ‐ ***y***_5_) shows that the latter is preferred at lower energies but reflects random cleavage at higher energies (right).

Strikingly, covalent bond cleavage, including phosphodiester backbone bond cleavage and that leading to the loss of nucleobases, was the lowest‐energy dissociation channel in CAD of the (Q + 3K)^4−^ quadruplex ions of RNA **1** (Figure 7). Two different factors can account for this phenomenon. First, the relatively low negative net charge involving three positive and seven negative charges minimizes the destabilizing effects of Coulombic repulsion and allows for strong hydrogen bonding networks within the quadruplex structure. Second, the increased number of phosphodiester moieties that are not deprotonated promotes backbone bond cleavage into ***c*** and ***y*** fragments [66, 67, 68]. To find out if the quadruplex structure is not just reflected in the ESI spectra but also in the products from covalent bond cleavage, we analyzed the data from CAD of the (Q + 3K)^4−^ quadruplex ions of RNA **1** in more detail. The loss of nucleobases (mostly guanine and some adenine) from otherwise intact quadruplex ions gave rise to the dominant products from covalent bond cleavage at energies up to ∼160 eV. The guanine bases likely originate from the 5′ G‐tetrad, the disassembly of which may be the first step in unraveling of the quadruplexes of RNA **1**, and the adenine bases can only originate from the 2 nt overhang (Figure 2). Above ∼160 eV, ***c*** and ***y*** fragments were the most abundant products from covalent bond cleavage, and, as also observed with CAD of other RNAs [23, 57, 66, 67], the yield of ***a*** and ***w*** fragments increased with increasing energy (Figure 7). When the ***c*** and ***y*** fragments are separated as originating from RNA backbone cleavage between tetrads and RNA backbone cleavage in the overhang, a clear trend can be observed. At lower energies, up to ∼80% of all ***c*** and ***y*** fragments result from cleavage in the overhang, despite the fact that they represent only two backbone cleavage sites of RNA **1** (40% out of the five cleavage sites in total), as opposed to the three cleavage sites between tetrads (60% out of the five cleavage sites in total). At higher energies, the fraction of ***c*** and ***y*** fragments resulting from cleavage in the overhang decreases to the 40% expected for random backbone cleavage; this finding is consistent with previous CAD studies of monomeric G‐quadruplexes of DNA [37]. Put another way, RNA backbone cleavage between tetrads is disfavored at lower energies available for dissociation, which is further evidence for the preservation of the native quadruplex structure in the gas phase.

## 4 Conclusions

The data presented here demonstrate that tetramolecular G‐quadruplex structures of RNA **1** and **2** with K^+^ and ^+^NH_4_ as central cations are sufficiently stable to survive transfer into the gas phase by native ESI, regardless of quadruplex ion net charge. For both RNA **1** and RNA **2**, the monomer‐quadruplex equilibrium present in solution was qualitatively reflected in the relative ion abundances in the ESI spectra. However, caution should be used when interpreting spectra of G‐quadruplex structures with ^+^NH_4_ as central cation. Unintended vibrational activation in the source region of the mass spectrometer can result in proton transfer from ^+^NH_4_ to the RNA, followed by loss of NH_3_, which destabilized the gaseous quadruplex structures of RNA **1** and RNA **2** with a net negative charge of five by ∼28% compared to those with K^+^ as central cations.

With both K^+^ and ^+^NH_4_ as central cations, we found that the gaseous G‐quadruplex structures of RNA **1** were ∼20% more stable than those of RNA **2**. This finding provides strong, additional evidence for the preservation of the quadruplex structures in the gas phase, as it reflects the contribution of the U‐tetrad and the additional central cation to the stability of the G‐quadruplexes of RNA **1**. Not surprisingly, the energy required for quadruplex ion dissociation in the gas phase steadily decreased with increasing net charge, which reflects the weakening of hydrogen bonding networks by Coulombic repulsion. Moreover, the products from noncovalent bond dissociation in the gas phase were different for different quadruplex ion net charges *n*. For *n* = 4–6, monomeric and trimeric RNA species were the most abundant, whereas for *n* = 7–8, monomeric and dimeric RNA species were dominant. This observation can be rationalized by the additional weakening of the interactions between the central cations and the nucleobases of the tetrads as the number of deprotonated sites on the RNA backbone increases. Nevertheless, for all *n*, dissociation into trimeric and monomeric species was the lowest‐energy channel for noncovalent bond dissociation, reflecting the preservation of a maximum number of interactions between the central cations and the nucleobases forming the central channel of the quadruplex. Although quadruplex ion stability in the gas phase decreases with increasing net charge, even the most highly charged quadruplex ions detected here, (Q + 3K)^8−^ at m/z 970, are stable and do not spontaneously dissociate, but rather require energy input for dissociation. Consistent with the fact that RNA is a polyanion, native MS of RNA in negative ion mode can produce ions with relatively high net charge without compromising the native structure. This is in contrast with data from native MS of proteins in positive ion mode, for which low net charge is often associated with native structures [69, 70].

Importantly, for quadruplex ions of RNA **1** with *n* = 4 and K^+^ as central cations, (Q + 3K)^4−^, the overall lowest‐energy dissociation channel was covalent bond dissociation rather than noncovalent bond dissociation, favoring products from RNA backbone cleavage in the overhang over those between the tetrads at lower energies. We attribute this phenomenon to the relatively balanced number of positive and negative charges at *n* = 4, which enforces strong electrostatic interactions in extended hydrogen bonding networks, minimizes Coulombic repulsion, and promotes facile RNA backbone bond cleavage. Taken together, the data presented here contribute to a better understanding of the stability of RNA interactions during and after desolvation, and thus the development of native MS for the characterization of RNA structure and binding [24, 27, 71, 72, 73].

## Supporting Information

Additional supporting information can be found online in the Supporting Information Section. **Supporting Fig. S1:** UV absorption, A, and dA/dT at 250, 260, and 295 nm of aqueous solutions of RNA **1** (solid black lines) and RNA **2** (dashed black lines) with potassium chloride (120 µM) and 200 mM HFIP at pH 5.5 as used for ESI experiments, RNA concentration was 95 µM. The heating curves were empirically found to fit functions containing a linear term in T and one or two sigmoidal terms in T; these fits and their derivatives are overlaid on the heating curves and their derivatives in red for RNA **1** and blue for RNA **2**. **Supporting Fig. S2:** MS spectra of 2 µM RNA **1** electrosprayed from aqueous solutions with potassium chloride (120 µM) and 200 mM HFIP (84 % RNA in Q) or 200 mM DFEA (68 % RNA in Q) or 200 mM TEAA (85 % RNA in Q). **Supporting Fig. S3:** Percentage stacked area plots show that the sequential loss of three (RNA **1**) and two (RNA **2**) NH3 molecules in CAD of (Q + 3NH4)^5‐^ ions of RNA **1** and RNA **2** (Q, solid circles) occurs at lower laboratory‐frame energies than those required for dissociation into monomer (M) and trimer (T) species. **Supporting Fig. S4:** Spectra from CAD of (Q + 3K)^4‐^, (Q + 3K)^5‐^, (Q + 3K)^6‐^, (Q + 3K)^7‐^ and (Q + 3K)^8‐^ ions of RNA **1** at the laboratory frame energies required for 50% quadruplex dissociation into monomeric (M), dimeric (D), and trimeric (T) species and products from covalent bond cleavage. **Supporting Table S1:** Percentage of RNA species observed in the spectra in Figure 2; M = monomer, D = dimer, Q = tetramer.

## Funding

This research was funded in whole or in part by the Austrian Science Fund (FWF) [grant DOI 10.55776/P36011 to K.B.], and the Österreichische Forschungsförderungsgesellschaft (Grant FO999912210).

## Conflicts of Interest

The authors declare no conflicts of interest.

## Supporting information

Supplementary Material

## Data Availability

The data that support the findings of this study are available from the corresponding author upon reasonable request.
