# Supplementary material for: Exploring RNA G‐Quadruplex Stability in the Gas Phase: Insights from Native Mass Spectrometry
Source: Chempluschem. 2025 Dec 10;91(1):e202500679. doi: 10.1002/cplu.202500679 (PMC12777508; doi:10.1002/cplu.202500679)
Supplement: Supplementary file 1 — Supplementary Material [file CPLU-91-e202500679-s001.pdf]

Exploring RNA G-Quadruplex Stability in the Gas Phase: Insights from Native Mass Spectrometry

Anna Ploner, Sarah Viola Heel, and Kathrin Breuker*

Institute of Organic Chemistry and Center for Molecular Biosciences Innsbruck (CMBI), University of Innsbruck, Innrain 80-82, 6020 Innsbruck, Austria

Table S1. Percentage of RNA species observed in the spectra in Figure 2; M = monomer, D = dimer, Q = tetramer.

RNA	central cation	species	% species [a]	% RNA [b]
**1**	K^+	M	28	9
		D	10	7
		Q	62	84
	$^+NH_4$	M	43	16
		D	0	0
		Q	57	84
**2**	K^+	M	75	47
		D	8	10
		Q	17	43
	$^+NH_4$	M	88	68
		D	4	6
		Q	8	26

[a] percentage of species, [b] percentage of RNA molecules in respective species.

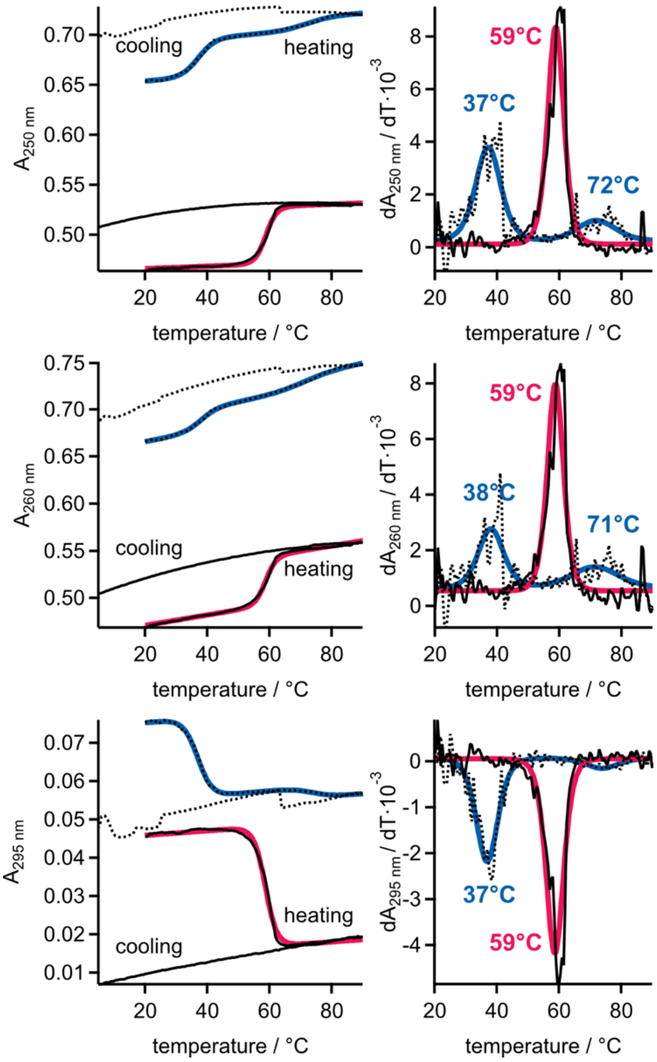

Figure S1. UV absorption, A, and dA/dT at 250, 260, and 295 nm of aqueous solutions of RNA **1** (solid black lines) and RNA **2** (dashed black lines) with potassium chloride (120 µM) and 200 mM HFIP at pH 5.5 as used for ESI experiments, RNA concentration was 95 µM. The heating curves were empirically found to fit functions containing a linear term in T and one or two sigmoidal terms in T; these fits and their derivatives are overlaid on the heating curves and their derivatives in red for RNA **1** and blue for RNA **2**.

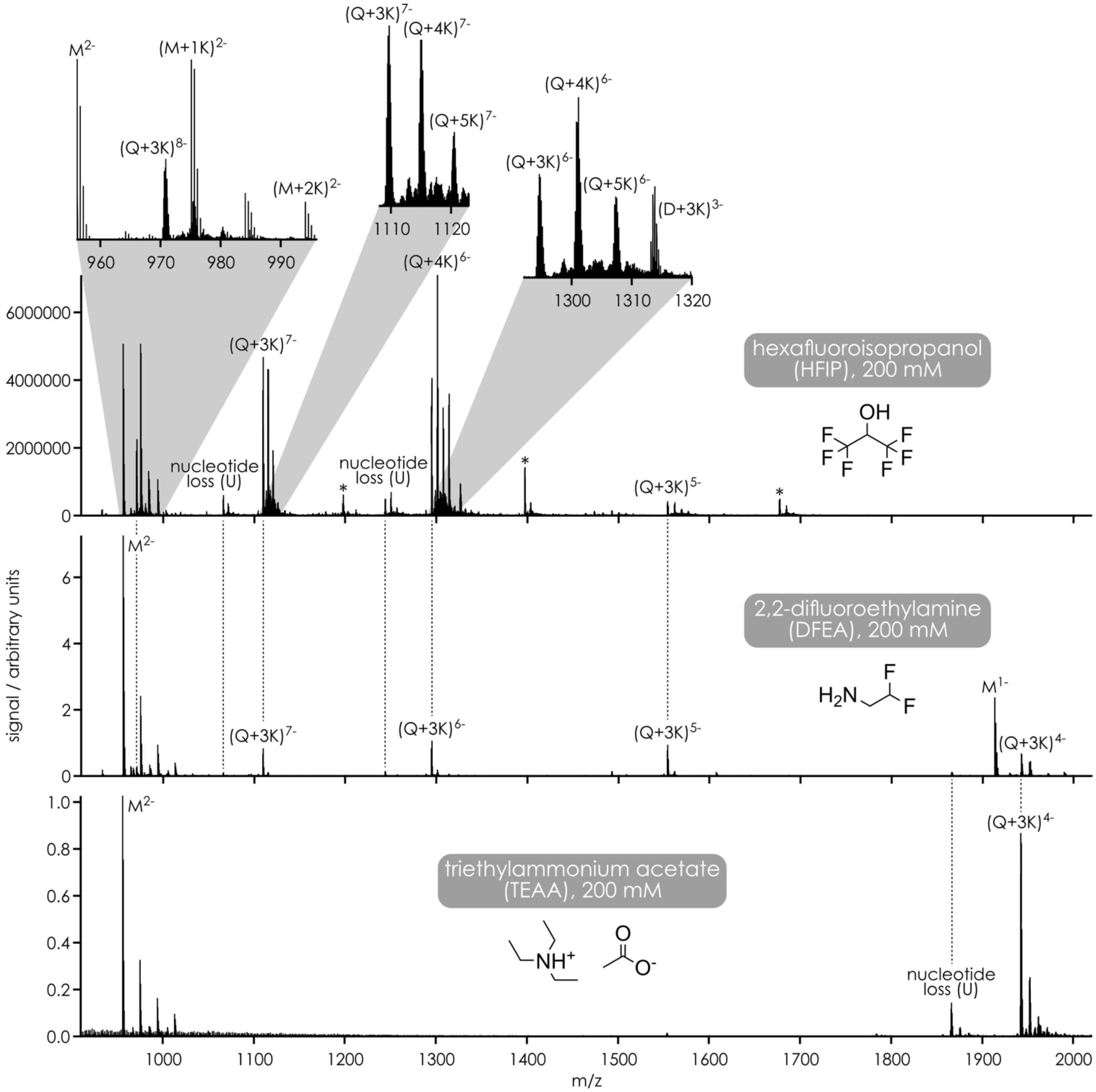

Figure S2. MS spectra of 2 µM RNA **1** electrosprayed from aqueous solutions with potassium chloride (120 µM) and 200 mM HFIP (84 % RNA in Q) or 200 mM DFEA (68 % RNA in Q) or 200 mM TEAA (85 % RNA in Q).

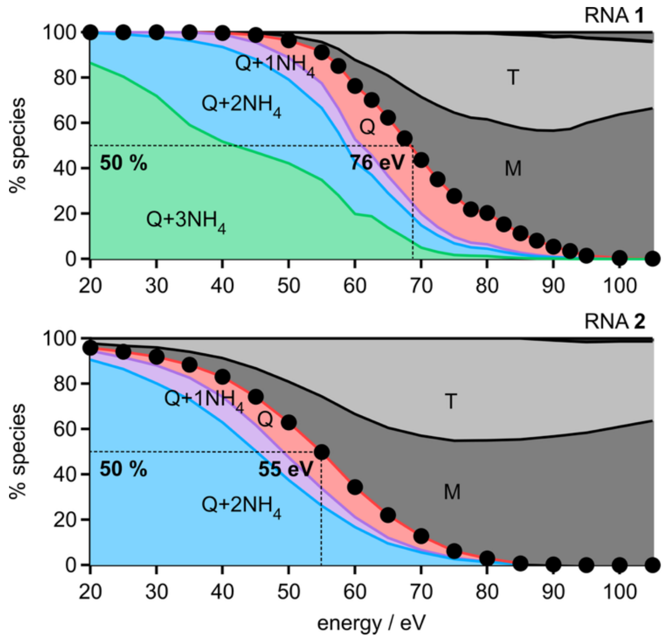

Figure S3. Percentage stacked area plots show that the sequential loss of three (RNA **1**) and two (RNA **2**) NH_3 molecules in CAD of $(Q + 3NH_4)^{5-}$ ions of RNA **1** and RNA **2** (Q, solid circles) occurs at lower laboratory-frame energies than those required for dissociation into monomer (M) and trimer (T) species.

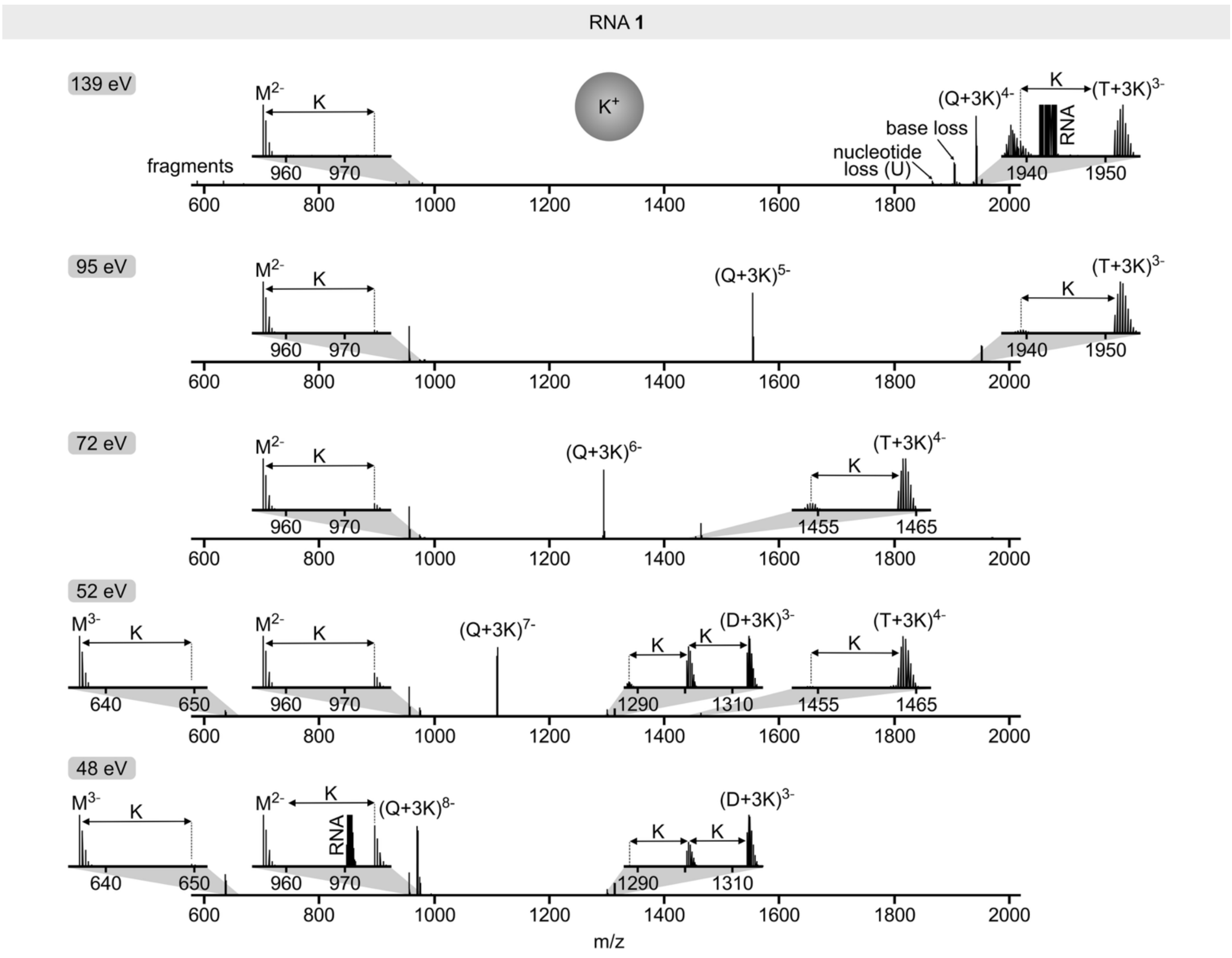

Figure S4. Spectra from CAD of $(Q + 3K)^{4-}$, $(Q + 3K)^{5-}$, $(Q + 3K)^{6-}$, $(Q + 3K)^{7-}$ and $(Q + 3K)^{8-}$ ions of RNA **1** at the laboratory-frame energies required for 50% quadruplex dissociation into monomeric (M), dimeric (D), and trimeric (T) species and products from covalent bond cleavage.
